# Impact of coach ethical leadership on athlete satisfaction and organizational commitment

**DOI:** 10.3389/fpsyg.2026.1856554

**Published:** 2026-07-21

**Authors:** Su Hang, Dongjin He, Kong Yaru

**Affiliations:** 1 Kaifeng University, Kaifeng, Henan, China; 2 Department of Physical Education, Guangdong University of Finance, Guangzhou, Guangdong, China; 3 Asia-Europe Institute, Universiti Malaya, Kuala Lumpur, Malaysia

**Keywords:** athlete satisfaction, Chinese traditionality, coach ethical leadership, organizational commitment, trust in coach

## Abstract

**Introduction:**

This study examines how coach ethical leadership influences the organizational commitment of Chinese competitive athletes by investigating the psychological roles of trust in coach and athlete satisfaction, as well as the moderating role of Chinese traditionality. Drawing on social learning theory and social exchange theory, the study proposes a moderated serial mediation model.

**Methods:**

A cross-sectional quantitative research design was employed. Data were collected from 468 competitive athletes in Nanjing, Wuhan, Xi’an, and Hangzhou, China. The hypothesized relationships were analyzed using partial least squares structural equation modeling in SmartPLS.

**Results:**

Coach ethical leadership was positively associated with athletes’ organizational commitment and trust in coach. Trust in coach was positively related to athlete satisfaction, which, in turn, was positively associated with organizational commitment. Trust in coach and athlete satisfaction sequentially mediated the relationship between coach ethical leadership and organizational commitment. Chinese traditionality negatively moderated the relationship between coach ethical leadership and trust in coach, such that this relationship was weaker among athletes with higher levels of traditionality. The conditional serial indirect effect of coach ethical leadership on organizational commitment was also weaker at higher levels of Chinese traditionality.

**Discussion:**

The findings demonstrate that ethical coaching contributes to athletes’ organizational commitment primarily by strengthening trust in coach and enhancing athlete satisfaction. By integrating ethical leadership, relational trust, athlete satisfaction, organizational commitment, and Chinese traditionality, this study extends sport leadership research and highlights the importance of culturally sensitive ethical coaching practices in Chinese competitive sport organizations.

## 1 Introduction

Ethical leadership has emerged as a growing concern in modern sport organizations, as coaches have a significant moral, psychological, and developmental role with athletes. Coaches have the power to influence team norms, expectations around training, access to opportunities, assessment of performances, and athletes’ sense of fairness, belonging, and their long-term commitment to their teams or sport organizations (Chelladurai and Saleh, 1980; Fletcher and Arnold, 2011; Jowett and Cockerill, 2003). This ethical aspect of coaching behavior is particularly relevant in a competitive sports context in which coaches have authority, are decisive in the selection and development of their athletes, and are confronted with high-performance stress (Constandt et al., 2020). Coaches who act fairly, honestly, caringly, responsibly, and in an ethical manner can build a more trustworthy and supportive environment in sport, while coaches who do not can erode athletes’ trust, satisfaction, and commitment to a team or sport organization (Brown et al., 2005; Mayer et al., 1995).

This study is primarily concerned with coach ethical leadership as a value-based leadership behavior that could potentially foster athletes’ commitment to their organization. Ethical Leadership involves modeling and communicating normatively appropriate behavior, both between people and within one’s own decision-making processes (Brown et al., 2005). In a sports setting, ethical leadership is exhibited in the way coaches treat athletes fairly, communicate clear expectations, do not show favoritism, safeguard the health and wellbeing of athletes, and demonstrate ethical behavior (Constandt et al., 2020). These behaviors are especially significant because athletes often seek feedback from coaches about their performance, look to coaches to assign them to the right roles, seek opportunities to compete, and look to coaches for development assistance (Jowett and Cockerill, 2003; Zhang and Chelladurai, 2013). Thus, athletes’ perceptions of coach ethical leadership could influence their attitudes toward the sport organization, as well as the amount of psychological attachment they form to the organization.

Based on the literature reviewed, organizational commitment in this study is defined as the affective attachment, identification, and desire to remain with the team, club, academy, university team, and/or sport organization. Although organizational commitment has been studied in employee samples, it is also relevant to competitive athletes because they belong to structured sport organizations with characteristics similar to those where employees work, including hierarchy, role expectations, training requirements, performance evaluation, and continuity of membership (Meyer and Allen, 1991; Meyer et al., 1993). Athletes are not employees, but they can form a mental attachment and loyalty to the organization or team they train and compete for. In addition, this construct of organizational commitment has been found to be applicable to the Chinese context in previous studies, further supporting its relevance to the current study (Cheng and Stockdale, 2003). Thus, understanding how ethical coaching relates to athlete commitment is crucial for their retention, team stability, and sustainable sport development.

Additionally, the study suggests that the effect of coach ethical leadership on organizational commitment is mediated by two consecutive psychological processes: trust in the coach and satisfaction with the coach. Trust in the coach is the athletes’ confidence that the coach is competent, reliable, fair, and benevolent (Mayer et al., 1995; McAllister, 1995; Rousseau et al., 1998). Trust is fundamental in coach–athlete relationships because athletes need to accept feedback, endure evaluation, depend on the coach’s decisions, and physically and psychologically open themselves up in training and competition (Jowett and Cockerill, 2003; Malloy et al., 2023). Ethical leadership can build trust, as coaches who consistently demonstrate that they will not misuse their power or be unfair to their players foster it (Brown et al., 2005). Trust, in turn, can improve athlete satisfaction, as athletes with a high degree of trust toward the coach tend to rate the training environment, interpersonal treatment, and team experience positively (Riemer and Chelladurai, 1998). By extension, athlete satisfaction can foster organizational commitment, as satisfied athletes tend to feel more committed to and stay within their sport organization (Meyer and Allen, 1991).

This study is set in the context of Chinese sport, where the relationship between coach and athlete can be influenced by personal leadership behaviors and culture. Previous studies on coach leadership in China have identified meaningful links between coach behaviors and athlete satisfaction and cohesion, which helps validate the importance of coach behavior in athlete outcomes in Chinese sports contexts (Zhu et al., 2024). Specifically, traditionalism in China may affect athletes’ perception of coaches’ authority and ethics. Chinese traditionalism is a manifestation of respect for authority, acceptance of hierarchical role obligations, and deference to senior persons (Farh et al., 1997). Athletes who exhibit high traditionalism may be more likely to attend to the formal authority and role legitimacy of the coach, while those with low traditionalism may attend more to the observable actions of the coach in determining trustworthiness. Therefore, the influence of Chinese traditionalism might affect the degree to which ethical leadership can lead to trust in the coach. This cultural boundary condition is significant because leadership effects might not be the same for every athlete, particularly in cultures that also place meaningful importance on hierarchy and authority in social and organizational life (Farh et al., 1997; Kirkman et al., 2009).

Although there is increasing interest in sport leadership, three gaps remain in the literature. First, many previous studies investigating coach leadership have examined general leadership behaviors, such as training and instruction, democratic behavior, autocratic behavior, social support, or positive feedback, rather than ethical leadership as a specific value-based predictor of athlete outcomes (Chelladurai and Saleh, 1980; Zhu et al., 2024). Second, research on the relationship between coaching and commitment has not adequately captured the psychology of the sequential process by which ethical coaching can lead to increased commitment. Trust and satisfaction have been investigated independently, but less research has focused on a sequential model where ethical leadership first leads to trust, followed by satisfaction, and then commitment (McAllister, 1995; Meyer and Allen, 1991; Riemer and Chelladurai, 1998). Third, culturally-based boundary conditions have received limited attention in Chinese sport leadership research. In sport leadership studies, moderators tend to be more demographic and sport-related than value-based cultural orientations like Chinese traditionality (Farh et al., 1997; Zhu et al., 2024).

To address these deficiencies, this study proposes and tests a moderated serial mediation model in the context of competitive athletes in China. Based on social learning theory and social exchange theory, the study explores whether coach ethical leadership is positively related to organizational commitment, whether there is sequential mediation between coach ethical leadership and trust in the coach, and between coach ethical leadership and athlete satisfaction (Bandura and Walters, 1977; Blau, 2017; Brown et al., 2005; Cropanzano and Mitchell, 2005), and whether Chinese traditionality moderates the relationship between coach ethical leadership and trust in the coach (Bandura and Walters, 1977; Blau, 2017). The model suggests that ethical leadership leads to a higher level of trust in the coach, ultimately leading to higher levels of athlete satisfaction, which, in turn, increases organizational commitment. The model also suggests that this serial indirect pathway is weaker for more traditional athletes, as the incremental value of coaches’ observable ethical conduct in establishing trust may be diminished when athletes are already strongly inclined to hierarchical authority.

The present work makes several contributions to the literature. In theory, it builds on ethical leadership research in the sport domain by investigating the role of coach ethical leadership as a predictor of athlete commitment (Brown et al., 2005; Constandt et al., 2020). It also incorporates principles from social learning theory and social exchange theory to account for the possible mechanisms by which ethical coaching can impact athletes: trust and satisfaction (Bandura and Walters, 1977; Blau, 2017; Cropanzano and Mitchell, 2005). Culturally, it represents the boundary conditions for the ethical leadership process (Farh et al., 1997; Kirkman et al., 2009). Practically, the study has implications for coach development, athlete management, and sport organization governance, as it demonstrates that ethical coaching is not only a moral expectation but also a relational and organizational resource that can contribute to athlete satisfaction and commitment.

## 2 Literature review and hypotheses development

### 2.1 Theoretical foundation

The study is based on two theories: social learning theory and social exchange theory. According to social learning theory, the relationship between coaches and athletes, through behaviors and values, can influence athletes’ trust in coaches. This view suggests that people learn proper conduct and evaluate authority figures based on observations of credible, powerful, behaviorally consistent role models (Bandura, 1986; Bandura and Walters, 1977). Coaches are also highly visible role models in competitive sport situations, as athletes are constantly attuned to coaches’ decisions, communication, opportunities, responses to errors, and conflict management (Jowett and Cockerill, 2003). A coach who acts fairly, honestly, caringly, and accountably provides clear behavioral cues to athletes that the coach is trustworthy and morally responsible (Brown et al., 2005; Constandt et al., 2020). Therefore, social learning theory can explain that coach ethical leadership will be positively related to trust in the coach.

This is complemented by social exchange theory, which helps to understand how trust and satisfaction can be converted to organizational commitment. According to social exchange theory, when people are treated fairly, respectfully, and supportively, they feel obliged to return this favor and develop positive attitudes toward others (Blau, 2017; Cropanzano and Mitchell, 2005). Ethical leadership may be viewed as a valuable relational resource within the coach–athlete relationship, as it indicates a coach’s regard for athletes and the responsible use of authority (Brown et al., 2005). Athletes can respond to this treatment by developing trust in the coach, having a more positive assessment of the sport experience, and becoming more emotionally committed to the team or sport organization (Hair et al., 2024; Mayer et al., 1995; McAllister, 1995; Riemer and Chelladurai, 1998). Thus, social exchange theory supports a positive relationship between ethical leadership and trust, trust and satisfaction, and satisfaction and organizational commitment.

The cultural dimension of traditionality is introduced through a boundary condition in the form of tradition. Traditionality is introduced as a culturally founded boundary condition. Traditionality is defined as endorsement of hierarchical role obligations, respect for authority, and deference to senior persons (Farh et al., 1997). In coach-led sport organizations, these values can impact the degree to which athletes rely on observable ethical behavior to determine the coach’s reliability. Less traditional athletes might judge trust more by what the coach actually does, rather than what they say, including being fair, transparent, and consistent in their moral behavior. The coach’s ethical leadership behavior might have less of an impact on trust for athletes with higher traditionality because they already feel that the coach is a legitimate authority figure. In this regard, the conceptualization of Chinese traditionality as a moderator between the first stage of coach ethical leadership and trust in coach is highly theoretically plausible.

### 2.2 Coach ethical leadership and organizational commitment

Ethical leadership of coaches should be positively related to athletes’ organizational commitment. Ethical coaches are characterized by integrity, fairness, accountability, and concern for athlete welfare (Brown et al., 2005). These behaviors might contribute to athletes viewing the sport organization as legitimate, supportive, and worthy of continued involvement. Athletes in competitive sports environments are often subject to performance judgments, selection, training stresses, and role assignments. The use of ethical coaching behavior could potentially increase an athlete’s identification with the team or organization and their emotional connection to it (Meyer and Allen, 1991; Meyer et al., 1993).

Theoretical background research indicates that commitment can be improved by ethical and value-based leadership, as followers tend to be loyal to organizations when leaders are ethical and behave fairly (Brown et al., 2005; Meyer and Allen, 1991). Additional research from the sport context suggests that athletes have a positive response to coaches’ perceived principled, supportive, and consistent behavior (Jowett and Cockerill, 2003; Malloy et al., 2023; Zhang and Chelladurai, 2013). Thus, coaching ethical leadership should directly influence increases in the organizational commitment of athletes.

*H1*: Coach ethical leadership is positively associated with athletes’ organizational commitment.

### 2.3 Coach ethical leadership and trust in coach

A primary relational product of ethical leadership is trust in the coach. Trust is usually characterized by a state of vulnerability resulting from the positive expectation of the other party’s intentions or actions (Mayer et al., 1995; Rousseau et al., 1998). Coaches play a key role in providing developmental feedback, access to competitions, training suggestions, and direction regarding decisions about an athlete’s role. This dependency requires the athlete to make decisions about the reliability, fairness, competency, and beneficence of the coach (McAllister, 1995). Ethical leadership offers a continual demonstration of the coach’s ethical use of authority and their lack of exploiting the athlete’s vulnerability (Brown et al., 2005).

In the social learning context, ethical coaches are seen as credible moral models because their behavior is congruent with their words and actions, which is consistent with the work of Mayer et al. (1995) and Brown et al. (2005). Athletes can judge and deduce the coach’s reliability based on the coach’s decision-making process, verbal communication, and care for the athlete’s wellbeing. When a coach’s impact on an athlete’s psychological experience is as great as it is in sport, trust is particularly crucial for cooperation and commitment (Jowett and Cockerill, 2003; Malloy et al., 2023). This suggests that coach ethical leadership is expected to increase trust in the coach.

*H2*: Coach ethical leadership is positively associated with athletes’ trust in their coach.

### 2.4 Trust in coach and athlete satisfaction

Trust in a coach is also expected to enhance athlete satisfaction. Athlete satisfaction reflects athletes’ affective and cognitive evaluation of their sport experience, including perceptions of coaching, training, team functioning, role use, and interpersonal treatment (Riemer and Chelladurai, 1998). When athletes trust their coach, they are more likely to interpret coaching feedback, training demands, and performance decisions as fair, developmental, and well intentioned. Trust reduces uncertainty and helps athletes feel psychologically safer in the sport environment (Mayer et al., 1995; McAllister, 1995).

In coach–athlete relationships, trust may make athletes more accepting of demanding training processes because they believe that the coach is acting in their best interest. Athletes who trust their coach may also feel more respected, supported, and valued. Prior work on coach–athlete relationships indicates that relational quality is closely linked to athlete experiences and outcomes (Jowett and Cockerill, 2003), while Chinese sport-leadership research confirms that coach leadership behaviors are associated with athlete satisfaction (Zhu et al., 2024). Therefore, trust in a coach is expected to be positively associated with athlete satisfaction.

*H3*: Athletes’ trust in coach is positively associated with athlete satisfaction.

### 2.5 Athlete satisfaction and organizational commitment

The satisfaction of the athletes is anticipated to contribute to organizational commitment. When athletes are content with their team, club, academy, or sport organization they view it as a meaningful and supportive environment (Riemer and Chelladurai, 1998). Coaching, interpersonal treatment, team processes, and performance development that are rated positively can help athletes’ emotional connection and commitment to the organization.

Commitment in sport organizations is not just about formal membership, but also about athletes’ sense of belonging, identification, and desire to continue. Affective commitment is particularly important because it reflects the emotional attachment to the organization (Meyer and Allen, 1991; Meyer et al., 1993). If athletes are content with their experience in their sport, they will be more inclined to form this affective attachment. Thus, there should be a positive relationship between athlete satisfaction and organizational commitment.

*H4*: Athlete satisfaction is positively associated with athletes’ organizational commitment.

### 2.6 Mediating mechanisms of trust in coach and athlete satisfaction

The first path of mediation suggested the following: Coach ethical leadership—Trust—Athlete satisfaction. Ethical leadership might not only affect athletes’ satisfaction by directly impacting their perceptions of fairness or morality, but it also needs to first build relational confidence in the coach. Athletes who are more positive about their training environment and sport experiences will be so if they trust the coach (McAllister, 1995; Riemer and Chelladurai, 1998). Thus, trust in the coach is a means of relating and is a way in which ethical coaching leads to satisfaction for athletes.

*H5*: Trust in coach mediates the relationship between coach ethical leadership and athlete satisfaction.

The second mediating pathway suggests that athlete satisfaction will lie between trust in the coach and organizational commitment. Whereas trust can generate confidence in the coach, commitment to the team/sporting organization is likely to grow when this trust becomes a positive overall evaluation of the athlete’s sport experience. Thus, satisfaction serves as a judge to connect relational trust with organizational attachment (Meyer and Allen, 1991; Riemer and Chelladurai, 1998). Athletes who are confident in their coach are more likely to be happy with their coach, and a happy athlete is more likely to feel loyal to his/her team/organization.

*H6*: Athlete satisfaction mediates the relationship between trust in coach and athletes’ organizational commitment.

### 2.7 Serial mediation of trust in coach and athlete satisfaction

Based on the study’s results, the researcher suggests that the coach’s ethical leadership indirectly affects organizational commitment by way of trust in the coach and satisfaction with the coach, as a mediation process, in order. This pathway aligns with social learning theory and social exchange theory. Ethical leadership offers visible moral cues, from which the coach may be seen as being fair and trustworthy, and thus demonstrates that he or she is a moral person that may be observed by athletes (Bandura, 1986; Brown et al., 2005). Athletes then make positive appraisals of their sport experiences, which in turn lead to a greater emotional commitment to the organization (Blau, 2017; Cropanzano and Mitchell, 2005; Mayer et al., 1995).

This serial process is a more complete process than a direct effect process. It posits that by first creating relational trust and then enhancing satisfaction with the sport experience, ethical coaching promotes organizational commitment. Thus, it is expected that the relationship between coach ethical leadership and organizational commitment will be mediated by trust in the coach and satisfaction with the coach, in sequence.

*H7*: Coach ethical leadership is indirectly associated with athletes’ organizational commitment through the sequential mediation of trust in coach and athlete satisfaction.

### 2.8 Moderating role of Chinese traditionality

Chinese traditionality is suggested to moderate the Coach Ethical Leadership—Trust in Coach relationship. There are two possible ways to specify this hypothesis: as moderation, not mediation. Traditionality is defined as the attitude to authority, acceptance of hierarchical role expectations, and deference to senior persons (Farh et al., 1997). Traditionality could affect athletes’ trust judgments in Chinese sport contexts where coaches have significant power over training, evaluation, and selection.

Athletes with lower traditionality might base their trust in the coach more heavily on the coach’s observable actions. Ethical leadership behaviors like fairness, transparency, care, and consistency could be particularly significant indicators of coach trustworthiness for these athletes (Brown et al., 2005; Mayer et al., 1995). Athletes, on the other hand, who are more traditional, might have already accepted the coach’s authority due to their position. This means that the positive impact of ethical leadership behavior on trust might be less for athletes with high traditionality. However, this does not mean it is irrelevant for highly traditional athletes. Instead, it implies that the trust-creating impact of ethical leadership is less behavior-dependent when trust in hierarchical leadership is already high (Farh et al., 1997; Kirkman et al., 2009).

*H8*: Chinese traditionality moderates the positive relationship between coach ethical leadership and trust in coach, such that the relationship is weaker when Chinese traditionality is higher.

### 2.9 Moderated serial mediation

In addition, Chinese traditionality should moderate the direct route from coach ethical leadership to trust in coach and thus condition the complete serial indirect route from coach ethical leadership to organizational commitment via trust in coach and athlete satisfaction. Specifically, the lower the level of traditionality, the more influential ethical leadership will be on trust in coach, which will directly result in increased athlete satisfaction and ultimately in increased organizational commitment. The stronger the traditionality of Chinese culture, the weaker should be the initial relationship between ethical leadership and trust, which in turn, should weaken the second-stage serial indirect effect.

This hypothesis helps to explain exactly where the moderation is found in the serial mediation model. Traditionality does not moderate all paths in the model; it only moderates the first stage of the path from coach ethical leadership to trust in coach. Thus, the conditional indirect effect refers to the path: coach ethical leadership → trust in coach → athlete satisfaction → organizational commitment. This aligns with the conditional process logic, in which moderation in an earlier stage of an indirect effect can lead to the conditionality of the overall indirect effect (Hayes, 2018).

*H9*: Chinese traditionality moderates the serial indirect relationship between coach ethical leadership and athletes’ organizational commitment through trust in coach and athlete satisfaction, such that the indirect effect is weaker when Chinese traditionality is higher because Chinese traditionality attenuates the first-stage path from coach ethical leadership to trust in coach.

**Figure 1:**
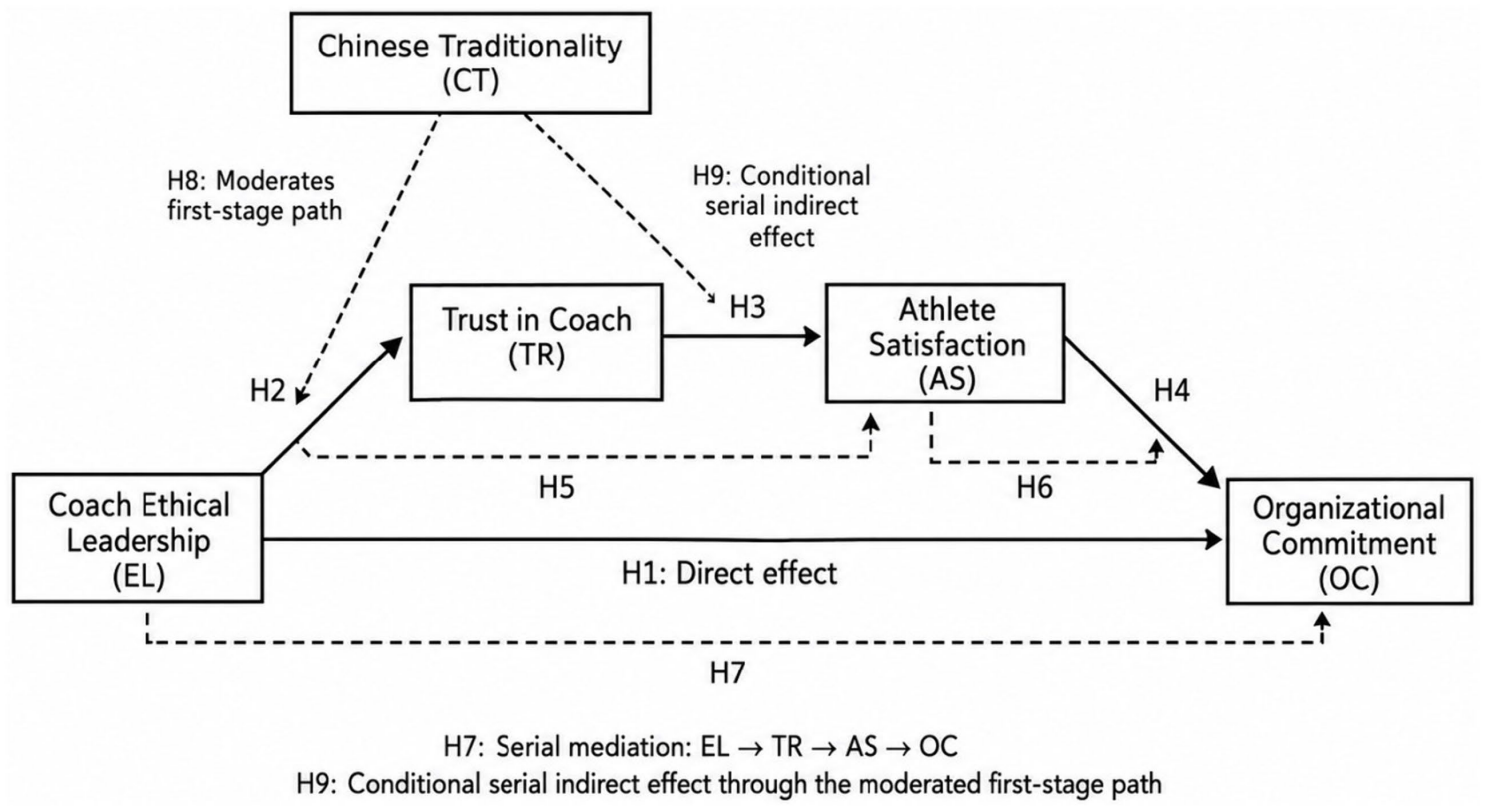
Preposed moderated serial mediation model.

The proposed conceptual model, presented in Figure 1, illustrates the direct relationship between coach ethical leadership and organizational commitment, the sequential mediating roles of trust in coach and athlete satisfaction, and the moderating role of Chinese traditionality on the first-stage path from coach ethical leadership to trust in coach.

## 3 Methods

### 3.1 Participants, population boundary, and setting

This study focused on competitive athletes from China within organized sport environments. These environments included university, provincial, sport club, or sport academy teams, where athletes were trained by an identifiable coach. This study was not designed to be representative of the entire population of all Chinese citizens, recreational exercisers, or informal sport participants. Instead, the population was limited to athletes actively involved in a structured sports organization with a coach, where coaching, practice, and selection were part of their experiences, and the playing club was an important part of their experience.

Only athletes from four Chinese cities participated: Nanjing, Wuhan, Xi’an, and Hangzhou. These cities were chosen to increase the variety of regions, rather than selecting the most studied mega-cities. In the East, the sport development contexts of Nanjing and Hangzhou are characterized by strong university development and club pathways. Wuhan provides a sport development context from a central Chinese collegiate and provincial setting, and Xi’an from a northwestern regional setting. Athletes were recruited from public university varsity teams, provincial sport institutes/sports schools, professional clubs’ academies/reserve teams, and private high-performance training centers within these cities.

The study required participants to have first-hand and long-term contact with coach-led sport organizations, so a multi-site purposive sampling strategy was used. To participate, individuals had to be 18 years of age or older, currently involved in organized competitive sport, coached by a coach, and have sufficient experience working with a coach and/or team to evaluate the coach’s ethical leadership, as well as their trust, satisfaction, and commitment with the coach and/or team. The sample size was appropriate for the proposed SmartPLS model; however, the sampling approach was not probabilistic. Therefore, the results should not be taken as representative of all Chinese athletes, but rather as evidence for the group of athletes competing in organized sports activities in selected groups in China.

### 3.2 Data collection procedure

Data were hand-collected using paper questionnaires completed during trainings and at team meeting rooms. These rooms were set up with the assistance of athletic administrators and team managers. Voluntary participation was explained, and trained research assistants introduced the study, emphasizing that there would be no observers to reduce the impact of authority and social desirability (Podsakoff et al., 2003). Respondents were requested to complete the questionnaire in the session itself and return it in envelopes placed in a collection box, which was only accessed by the research team. Each questionnaire was printed with a non-identifying numeric code to trace distribution and returns. To maintain anonymity, a tracking sheet with only information on whether a coded questionnaire was distributed and returned was created and stored separately from the completed forms.

### 3.3 Instrument adaptation and translation

This questionnaire was adapted to the coach-athlete setting and based on validated scales. Words in the Ethical Leadership Scale were changed to be coach-related rather than leader/supervisor-related. The trust scale was modified to specifically address trust in the coach. The questions about athlete satisfaction and organizational commitment were designed to elicit the athlete’s experience with their team, club, academy, and/or sport organization. Chinese traditionality items were preserved, and their traditional meanings were maintained within the culture.

The original instruments, developed in an organizational or sport context in English, were translated and back-translated to ensure linguistic and conceptual equivalence. The English items were translated into Chinese by two bilingual researchers. Secondly, two independent bilingual experts back-translated the Chinese version into English. Third, a comparison meeting was held to address differences between the original and back-translated versions. Where necessary, changes were made to items to enhance semantics, cultural appropriateness, and sport-context relevance. Cross-cultural scale adaptation is commonly performed by back-translation as it enables detecting meaning differences between source and target languages (Brislin, 1970).

The adapted questionnaire was pilot tested by three scholars in sport management and two organizational psychologists with experience in sport development in China to enhance content validity. They reviewed the clarity and understandability of the items for athletes, the appropriateness of the coach-related items, and the conceptual distinctiveness of the study constructs. A pilot test was then performed on 32 athletes not included in the final sample. The pilot participants indicated that the items were clear, understandable, and relevant to their sport context. At this stage, no items were removed; some minor wording adjustments were made to decrease word ambiguity and improve readability.

All the constructs were set to reflective. This specification was suitable because the indicators were used as expressions of the underlying constructs instead of as components that create or constitute the constructs. Athletes’ perceptions of fairness, integrity, and principled decision-making were considered a reflection of the coach’s ethical leadership, while their evaluation of sport-related experiences was treated as a reflection of athletes’ satisfaction. The results of the measurement model also supported this, as they showed acceptable indicator reliability and internal consistency to the authors, and high convergent and discriminant validity.

### 3.4 Measures

All constructs were assessed with scales validated for the coach–athlete/sport–organization setting. All English language measures were translated and back-translated in keeping with the above-mentioned procedures, as the study was conducted with Chinese athletes. Where required, items were also modified to be more sport-specific, using the terms “coach,” “team,” “club,” “academy” or “sport organization” for terms generally used in the workplace but applicable to sport. Responses were recorded on a seven-point Likert-type scale (1 = strongly disagree to 7 = strongly agree), unless otherwise stated. A higher score indicated a higher level of the respective construct.

The 10-item Ethical Leadership Scale (ELS) developed by Brown et al. (2005) was used to measure coach ethical leadership. To make the scale applicable to a coaching situation, “leader” was changed to “coach.” This measure reflects athletes’ perceptions of their coach’s fairness, integrity, ethical communication, and principled decision-making. Adapted items include “My coach sets an example on doing things the right way in regards to ethics” and “My coach makes fair and balanced decisions.”

McAllister’s (1995) interpersonal trust scale was used to assess trust in the coach. The scale originally consisted of cognition-based and affect-based trust dimensions and was modified to focus on athletes’ trust in their coach. Cognition-based trust is defined as trust based on the coach’s competence, reliability, and responsibility, while affect-based trust involves emotional trust and a sense of care from the coach to the athlete. Sample adapted items include “I can count on my coach not to place me at a disadvantage because of careless decisions,” and “I have a sharing relationship with my coach; I can share my ideas and feelings with them freely.”

The Athlete Satisfaction Questionnaire developed by Riemer and Chelladurai (1998) was used to determine athlete satisfaction. The ASQ is a multidimensional sport-specific questionnaire that measures athletes’ satisfaction with various components of the sport experience (coaching, team functioning, performance processes, and personal treatment). Items presented were linked to the current team the athlete is playing for, club, academy, or sporting body. Some of the items that may be included are “How satisfactory is it to be a member of a team that can be the best at the sport?” and “How satisfactory is it that your abilities are used during the season?”

The affective commitment scale from Meyer et al.’s (1991, 1993) three-component model of organizational commitment was used to measure organizational commitment. Affective commitment was chosen because it was most consistent with the present study’s focus on athletes’ emotional attachment, identification, and desire to remain involved in their team or sport organization. Items were adapted to the sports situation. Sample adapted items include “I feel a strong sense of belonging to my team/club” and “I feel emotionally attached to my team/club.”

For Chinese traditionality, the traditionality scale for Chinese cultural and organizational studies by Farh et al. (1997) was used. The scale measures the degree to which individuals agree that they have role obligations to those above them, feel obligated to respect authority, and defer to older individuals. This construct has been included as an individual’s cultural value orientation. Sample items include “The best way to avoid mistakes is to follow the instructions of senior persons” or “When people are in dispute, they should ask the most senior person to decide who is right.”

Gender, age, city, sport type, and organization pathway were used as control variables. These variables were selected because factors related to the athlete’s background and the sport setting can impact satisfaction, trust, and commitment. The control variables, however, were not integral to the theoretical model and were only included as a test of the robustness of the hypothesized relationships (See Appendix, Table A1).

### 3.5 Ethical considerations

This study received ethical approval from Guangdong University of Finance, China (approval number: GDUF-PE-REC-2025-071). Participation in the study was completely voluntary, and respondents were informed they could withdraw at any time without penalty. The consent statement also ensured that data would be analyzed and reported only in summary form and would not be shared with coaches, administrators, or any other officials of the institution. Completed questionnaires were kept securely while in the field and then transferred to password-protected and encrypted files, accessible only to the research team. These practices are compatible with best-practice recommendations that focus on ensuring no undue influence and preserving confidentiality when conducting field surveys (Hair et al., 2024).

### 3.6 Sample size estimation

The *a priori* type of power planning was used instead of rule-of-thumb heuristics during sample size planning. Despite PLS-SEM’s robustness with smaller samples, recent methodological literature emphasizes that an adequate sample size is required to achieve stable parameter estimation, indirect effects bootstrapping, and sufficient power to detect small effects, particularly in models with mediation and interaction terms (Hair and Alamer, 2022; Kock, 2015). Since the 10-times rule has been criticized as generating imprecise and occasionally underpowered estimates, power-based planning has been suggested for modern-day PLS-SEM (Jhantasana, 2023). We set the desired power = 0.95, 5 with a small effect size of f 2 = 0.02, and 7 with 8 = 0.05. Using G*power (Faul et al., 2009), we obtained a recommended sample size of about 400 for the present moderated serial mediation model.

### 3.7 Response rate and data screening

The total sample size of 563 was based on a power-based sample size calculation, accounting for potential non-response typical of survey-based research. Out of these, 497 questionnaires were received. Following data quality screening, 29 cases were discarded, leaving 468 valid cases for final analysis. Questionnaires were discarded for a variety of reasons, such as (a) a high percentage of item non-response (more than 10% of items missing); (b) low accuracy on attention checks, as measured through items not answered in the instructed manner (less than 50% accuracy); (c) straight-lining or patterned responses across multiple constructs (less than 50% accuracy); (d) internally inconsistent responses to closely related or adjacent items (less than 50% accuracy); and (e) on-site completion time that was too short (less than 10 min). This screening method aligns with general quality control practices in SEM survey research to safeguard measurement validity and remove noise in structural estimates (Hair et al., 2024).

### 3.8 Sample characteristics

Table 1 reports the socio-demographic profile of the final sample (N = 468) and documents the organization segments represented across the four cities.

**Table 1:** Sample socio-demographic characteristics and organization segments (*N* = 468).

Characteristic	Category	Frequency (*n*)	Percentage (%)	Organization segment included (examples)
Gender	Male	260	55.6	University varsity; provincial sport institute/school; club academy; private training center
	Female	208	44.4	University varsity; provincial sport institute/school; club academy; private training center
Age	18–20	156	33.3	—
	21–23	198	42.3	—
	24+	114	24.4	—
City	Nanjing	120	25.6	Public universities with varsity teams; club youth/reserve squads
	Wuhan	118	25.2	Comprehensive universities; provincial sport institutes/training bases
	Xi’an	110	23.5	Universities; provincial sport schools; regional club programs
	Hangzhou	120	25.6	Universities; club academies; private high-performance training centers
Sport type	Team sports	278	59.4	Football/basketball/volleyball and comparable team programs
	Individual sports	190	40.6	Badminton/table tennis/athletics and comparable individual programs
Organization pathway	University/Collegiate	210	44.9	University athletic departments; intercollegiate teams
	Provincial institute/Sport school	118	25.2	Provincial sport schools; specialized training centers
	Professional club academy/Reserve	92	19.7	Professional club youth/reserve development systems
	Private training academy	48	10.3	Private high-performance training organizations

The final age group was collapsed into 24+ to improve frequency balance across age categories. This grouping is also defensible because international demographic conventions commonly treat youth as extending up to age 24, although age classifications vary across organizations and national contexts.

## 4 Findings

This section presents the empirical findings of the study. Following methodological recommendations for PLS-SEM, the analysis proceeded in three stages. First, the measurement model was assessed to establish reliability, convergent validity, discriminant validity, collinearity, and data quality. Second, the structural model was evaluated to test the direct, mediating, moderating, and moderated serial mediation hypotheses. Third, predictive relevance and robustness checks were examined to strengthen confidence in the findings. The hypotheses are discussed separately to clearly indicate whether each one was supported.

### 4.1 Measurement model assessment and data health

The reflective measurement model was evaluated using SmartPLS prior to testing the structural relationships. The results revealed that all Cronbach’s alpha and composite reliability values were higher than the recommended value of 0.70, indicating good internal consistency reliability. Convergent validity was also confirmed, as the average variance extracted values for all constructs were greater than 0.50. The results show that the indicators successfully reflected the latent constructs.

Discriminant validity was examined through the Fornell–Larcker criterion and the heterotrait-to-monotrait ratio of correlations. The square root of the AVE for each construct was higher than its correlation with other constructs, indicating that the Fornell–Larcker criterion was met. Furthermore, all HTMT values were lower than the conservative value of 0.85, indicating empirical distinctiveness of the constructs. The variance inflation factor values based on full collinearity were lower than 3.30, which indicated that there was not a serious common method bias threat to the interpretation of the results. The values for inner VIF were also acceptable, meaning that multicollinearity did not affect the estimates of the structural models.

Explanatory and predictive power were also satisfactory for the model. The *R*^2^ values suggested that the model accounted for meaningful variance in trust in coach, athlete satisfaction, and organizational commitment. Furthermore, *Q*^2^ values were positive for the endogenous constructs, indicating predictive relevance. All these results combined to show that the dataset and measurement model were appropriate for hypothesis testing (see Table 2).

**Table 2:** Measurement and structural diagnostics.

Construct/Indicator	*α*	CR	AVE	FL	HTMT	Full VIF	Inner VIF	*R* ^2^	*Q* ^2^	Main *f*^2^
Ethical leadership (EL)	0.91	0.93	0.63	Yes	0.72	2.12	—	—	—	0.48
Trust in coach (TR)	0.90	0.92	0.66	Yes	0.78	2.28	2.41	0.44	0.29	0.51
Athlete satisfaction (AS)	0.88	0.91	0.60	Yes	0.78	2.45	1.86	0.34	0.21	0.40
Organizational commitment (OC)	0.89	0.93	0.69	Yes	0.66	2.19	2.05	0.56	0.33	0.04
Chinese traditionality (CT)	0.85	0.90	0.62	Yes	0.24	1.76	—	—	—	0.03
Model-level indicator	—	—	—	—	—	—	—	—	—	—
SRMR	—	—	—	—	—	—	—	—	—	0.057

*α*, Cronbach’s alpha; CR, composite reliability; AVE, average variance extracted; FL, Fornell–Larcker criterion satisfied; HTMT, highest heterotrait-to-monotrait ratio involving the construct. Full collinearity VIF values below 3.30 suggest that common method bias is unlikely to represent a serious concern. *Q*^2^ values greater than zero indicate predictive relevance. *f*^2^ values of approximately 0.02, 0.15, and 0.35 represent small, medium, and large effect sizes, respectively. Control variables were examined; however, because they were collectively non-significant, they were omitted from the main table for parsimony.

Because athletes were recruited through teams, academies, and sport organizations, the potential non-independence of observations was also assessed. Intraclass correlation coefficients were calculated for the main endogenous constructs in team-based sessions for which grouping information was available. The ICC (1) values were low for trust in coach (0.021), athlete satisfaction (0.018), and organizational commitment (0.027). In addition, all design effects were below the commonly applied threshold of 2.0, indicating that clustering was unlikely to cause serious violations of the independence assumption. Accordingly, multilevel modeling was not employed in the primary analysis. Nevertheless, team-based recruitment is acknowledged as a methodological limitation, and future studies should collect more complete coach- and team-level identifiers to enable the application of multilevel SEM. Assessing non-independence through ICC values is a common diagnostic procedure in nested organizational and sport-related data (Bliese, 2000).

#### 4.1.1 Summary of data quality

Overall, the diagnostic evidence indicates that the dataset was appropriate for hypothesis testing. The measurement model demonstrated satisfactory reliability and convergent validity, while discriminant validity was confirmed using both the Fornell–Larcker criterion and HTMT ratios. Common method bias did not appear to be severe, and the results for multicollinearity and predictive relevance were within acceptable limits. Taken together, these findings support proceeding to the structural model assessment and hypothesis testing in SmartPLS.

### 4.2 Hypotheses testing (direct effect)

H1 predicted a positive relationship between coach ethical leadership and athletes’ organizational commitment. This hypothesis was confirmed by the results. In terms of organizational commitment, coach ethical leadership had a positive and statistically significant effect (*β* = 0.12, *t* = 2.45, *p* = 0.014, 95% CI [0.024, 0.215]). The finding suggests that athletes’ perception of their coaches’ characteristics of being fair, ethical, honest, and principled was modestly associated with athletes’ emotional attachment to their sport organization. Hence, H1 was supported.

H2 suggested that the greater the athletes’ trust in their coach, the more positive the relationship between coach ethical leadership and athletes’ trust in their coach. This hypothesis was supported by the results. Coach ethical leadership had a positive and statistically significant effect on trust in coach (*β* = 0.60, *t* = 15.20, *p* < 0.001, 95% CI [0.523, 0.677]). This was the strongest direct relationship in the model, suggesting that the ethical behaviors of coaches are directly related to athletes’ confidence in their coach’s ethical fairness, reliability, competence, and benevolence. Thus, H2 was supported.

H3 stated that the relationship between athletes’ trust in their coach and their satisfaction would be positive. This hypothesis was confirmed. Trust in coach had a positive and statistically significant effect on athlete satisfaction (*β* = 0.58, *t* = 13.10, *p* < 0.001, 95% CI [0.493, 0.666]). This indicates that athletes who relied on their coach were more likely to have positive perceptions of coaching abilities, training conditions, interpersonal relationships, and team experiences. Thus, H3 was supported.

H4 hypothesized that athletes’ satisfaction would be positively related to athletes’ organizational commitment. This was confirmed by the results obtained. Organizational commitment (*β* = 0.55, *t* = 12.40, *p* < 0.001, 95% CI [0.462, 0.638]) was found to be positively and statistically significantly affected by athlete satisfaction. The results revealed that those athletes who were more satisfied with their sport experience had a higher likelihood of experiencing emotional attachment and identification with their sport organization, academy, club, and/or team. Thus, H4 was supported.

### 4.3 Mediation effects

H5 suggested that trust in coach would serve as a mediator between coach ethical leadership and athlete satisfaction. This hypothesis was confirmed by the bootstrapping results. The indirect effect of coach ethical leadership on athlete satisfaction via trust in coach was positive and significant (*β* = 0.35, *t* = 8.96, *p* < 0.001, 95% CI [0.276, 0.424]). Partial mediation is suggested by the VAF value of 71.4% for this pathway. This suggests that coach ethical leadership had a direct relation with athlete satisfaction, as well as an indirect relation via the athlete’s trust in coach, with a significant portion of the link going through the athlete’s trust in coach. Thus, H5 was endorsed.

H6 posited a mediating effect of athlete satisfaction between trust in coach and organizational commitment of athletes. This hypothesis was confirmed by the results. The indirect effect of trust in coach on organizational commitment through athlete satisfaction was positive and statistically significant (*β* = 0.32, *t* = 7.84, *p* < 0.001, 95% CI [0.244, 0.397]). The VAF value was 64.0%, suggesting partial mediation. The finding implies that trust in the coach enhanced athletes’ organizational commitment, in part because trust enhanced athletes’ satisfaction with their sport experiences. Thus, H6 was supported.

H7 suggested that organizational commitment of athletes was indirectly related to coach ethical leadership by the sequential mediation of trust in coach and athlete satisfaction. This hypothesis was supported by the results. The indirect effect of coach ethical leadership on organizational commitment, mediated by trust in coach and athlete satisfaction, was significant (*β* = 0.19, *t* = 6.85, *p* < 0.001, 95% CI [0.136, 0.244]). The overall VAF for the entire serial pathway was 61.3%, which represents partial serial mediation. This finding shows that the coach’s ethical leadership was linked to higher levels of trust in the coach, which was linked to higher athlete satisfaction with the coach, which in turn was linked to higher organizational commitment. Hence, H7 was supported.

### 4.4 Moderating effect of Chinese traditionality

H8 hypothesized that the positive association between coach ethical leadership and trust in coach would be weakened when Chinese traditionality was increased. This hypothesis was confirmed by the results. The interaction between coach ethical leadership and Chinese traditionality was negative and statistically significant in predicting trust in coach (*β* = −0.10, *t* = 2.98, *p* = 0.003, 95% CI [−0.166, −0.035]).

This interaction was further understood using simple slope analysis. The positive relationship between coach ethical leadership and trust in coach was strongest at low levels of Chinese traditionality (*β* = 0.70, *t* = 13.84, *p* < 0.001), moderate at the mean level of Chinese traditionality (*β* = 0.60, *t* = 15.20, *p* < 0.001), and weakest at high levels of Chinese traditionality (*β* = 0.50, *t* = 9.74, *p* < 0.001). The association of coach ethical leadership with trust in coach was thus still positive at all Chinese traditionality levels, but weakened with a rise in traditionality. So, H8 was supported.

### 4.5 Moderated serial mediation

**Figure 2:**
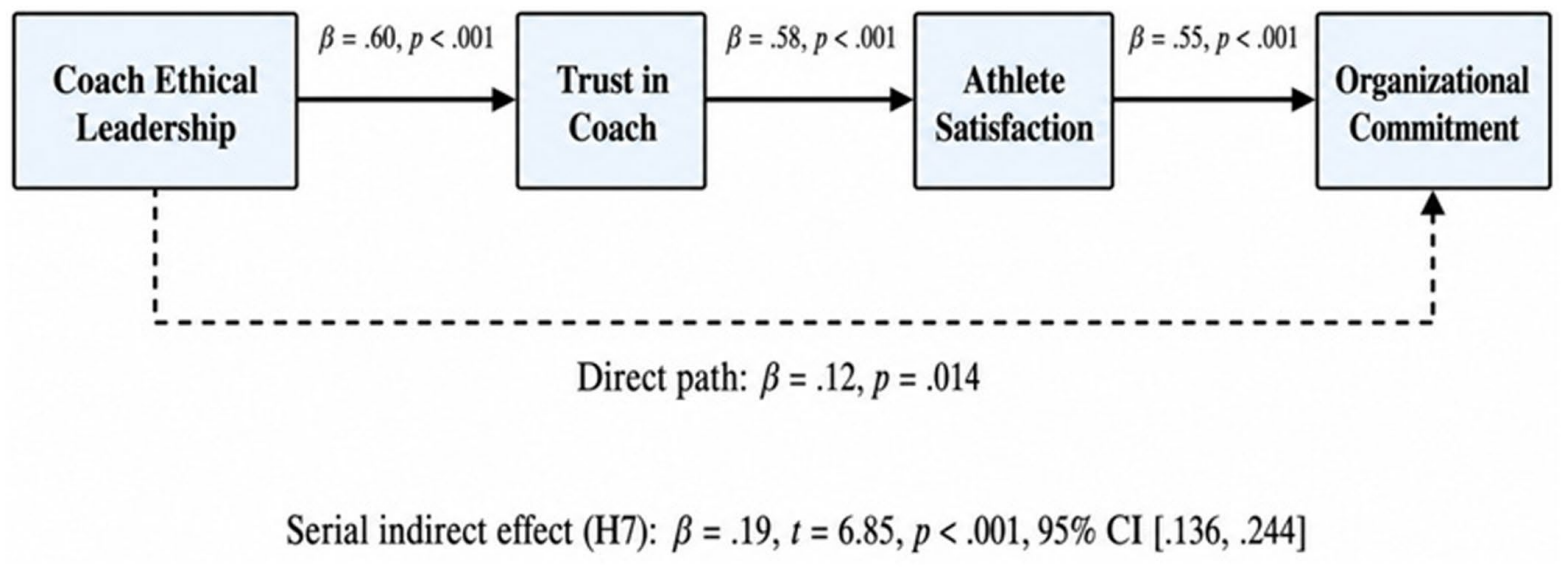
Serial mediation pathway for H7.

**Figure 3:**
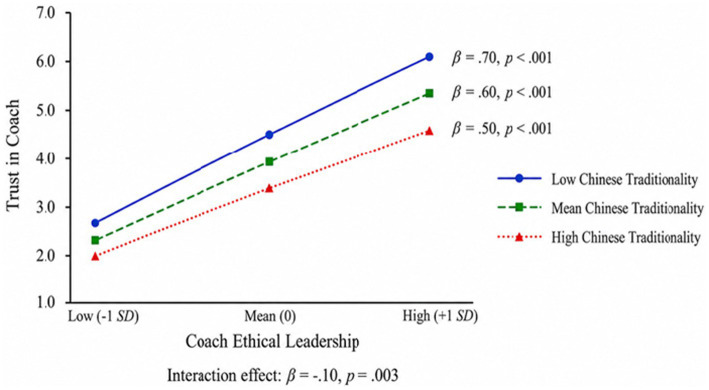
Moderating effect of Chinese traditionality on the relationship between coach ethical leadership and trust in coach. The positive association between coach ethical leadership and trust in coach was weaker at higher levels of Chinese traditionality. Simple slopes were significant at low traditionality (*β* = 0.70, *p* < 0.001), mean traditionality (*β* = 0.60, *p* < 0.001), and high traditionality (*β* = 0.50, *p* < 0.001), but the slope decreased as traditionality increased.

Based on this, H9 hypothesized a serial indirect effect of coach ethical leadership on organizational commitment via trust in coach and athlete satisfaction, which would be buffered by Chinese traditionality. Specifically, the hypothesis posited that the serial indirect effect would be weaker when Chinese traditionality was greater, due to the moderating effect of Chinese traditionality on the first-stage path from coach ethical leadership to trust in coach. This hypothesis was confirmed by the results (see Tables 3, 4; Figures 2, 3).

**Table 3:** Hypotheses testing results.

Hypothesis	Path/Effect	*β*	*t*	*p*	95% CI	Decision
H1	EL → OC	0.12	2.45	0.014	[0.024, 0.215]	Supported
H2	EL → TR	0.60	15.20	<0.001	[0.523, 0.677]	Supported
H3	TR → AS	0.58	13.10	<0.001	[0.493, 0.666]	Supported
H4	AS → OC	0.55	12.40	<0.001	[0.462, 0.638]	Supported
H5	EL → TR → AS	0.35	8.96	<0.001	[0.276, 0.424]	Supported
H6	TR → AS → OC	0.32	7.84	<0.001	[0.244, 0.397]	Supported
H7	EL → TR → AS → OC	0.19	6.85	<0.001	[0.136, 0.244]	Supported
H8	EL × CT → TR	−0.10	2.98	0.003	[−0.166, −0.035]	Supported
H9	Moderated serial mediation index	−0.032	2.75	0.006	[−0.055, −0.010]	Supported

EL, ethical leadership; TR, trust in coach; AS, athlete satisfaction; OC, organizational commitment; CT, Chinese traditionality. Confidence intervals are based on 5,000 bootstrapped resamples.

**Table 4:** Mediation assessment using bootstrap and VAF.

Mediation pathway	Direct effect	Indirect effect	Total effect	VAF	Mediation type
EL → TR → AS	0.14	0.35	0.49	71.4%	Partial mediation
TR → AS → OC	0.18	0.32	0.50	64.0%	Partial mediation
EL → TR → AS → OC	0.12	0.19	0.31	61.3%	Partial serial mediation

VAF, indirect effect/total effect × 100. VAF values between 20 and 80% indicate partial mediation. The direct effects were retained during the mediation assessment to avoid overstating the indirect pathways.

### 4.6 Predictive and robustness assessment

In addition to the explanatory path coefficients, PLSpredict was carried out with 10 folds and 10 repetitions to strengthen the evaluation of the model (Shmueli et al., 2019). The predictive relevance of the model was indicated by all positive *Q*^2^_predict values for the endogenous constructs. Most indicators of the endogenous constructs had lower prediction errors for the PLS-SEM model compared to the linear benchmark model. In particular, the PLS-SEM had a lower RMSE in 10 of 11 trust in coach indicators, 46 of 56 athlete satisfaction indicators, and 5 of 6 organizational commitment indicators. These results indicate medium predictive power for the model. If the researcher wants to show predictive performance, rather than just statistically explanatory results in the in-sample data, then PLSpredict is recommended for use in PLS-SEM (Hult et al., 2018) (see Table 5).

**Table 5:** PLSpredict summary.

Endogenous construct	*Q*^2^_predict	Indicators with lower RMSE than LM	Predictive conclusion
Trust in coach	0.291	10/11	Medium predictive power
Athlete satisfaction	0.224	46/56	Medium predictive power
Organizational commitment	0.318	5/6	Medium predictive power

A sensitivity analysis was conducted using the Gaussian copula approach to assess potential endogeneity. The primary theoretically relevant predictor relationships were first examined and found to be non-normally distributed, after which the corresponding Gaussian copula terms were generated and included in the model. None of the copula terms reached statistical significance: GC ethical leadership → trust in coach (*β* = 0.026, *p* = 0.401), GC trust in coach → athlete satisfaction (*β* = −0.019, *p* = 0.542), and GC athlete satisfaction → organizational commitment (*β* = 0.031, *p* = 0.338). The inclusion of these terms did not materially change the substantive path coefficients. Although causal inference remains constrained by the cross-sectional design, the results suggest that endogeneity is unlikely to have meaningfully biased the main findings. The Gaussian copula procedure is widely used as a sensitivity technique for assessing potential endogeneity concerns in structural equation modeling.

## 5 Discussion

The goal of this study was to investigate the relationship between coach ethical leadership and athletes’ organizational commitment and to understand the psychological and cultural processes leading to this relationship. Based on the concepts of social learning theory and social exchange theory, the study hypothesized that coach ethical leadership would increase organizational commitment by increasing trust in coach and athlete satisfaction. Chinese traditionality, a cultural boundary condition, was also explored in this study to provide insights into the relationship between coach ethical leadership and trust in coach at the first stage. In general, the results support the hypothesized moderated serial mediation model and help to explain the ethical leadership process in the competitive sports sector.

### 5.1 Coach ethical leadership as a moral and relational resource in sport organizations

This positive relationship between coach ethical leadership and organizational commitment suggests that athletes will be more committed to their team, club, academy, or sport organization if they believe their coach to be ethical, fair, honest, responsible, and consistent. This result supports the ethical leadership theory that posits that leaders positively affect followers not just by means of their technical expertise or direction of performance, but also by their moral behavior, fairness, and principled decision-making (Brown et al., 2005). This relationship is particularly significant in the sport context where coaches can sometimes be responsible for the provision of training opportunities, playing time, role allocations, selection, and developmental feedback. Thus, ethical coaching behavior becomes an important indicator for athletes to judge the organization’s value for loyalty and participation.

This discovery builds on the work of previous research in sport leadership in two significant ways. First, past research has tended to be more general, for example, on training and instruction, democratic behavior, social support, positive feedback, or autocratic behavior (Chelladurai and Saleh, 1980; Zhu et al., 2024). The present study indicates that ethical leadership is not just another generic leadership style but rather a value-based coaching style that influences athletes’ attitudes toward their organizations. Second, the impact of coach ethical leadership on organizational commitment was relatively small, in comparison to the impact through trust and satisfaction. This indicates that commitment is not a given when ethical leadership is present. Instead, it seems that ethical leadership establishes the relational and evaluative dynamics that nurture commitment. This is crucial as it takes one step further than a direct effect explanation and demonstrates that athletes’ commitment is a result of a series of psychological responses.

Ethical coaches are visible moral role models observed and interpreted by athletes from a social learning theory perspective (Bandura, 1986; Bandura and Walters, 1977). Athletes learn from coaches’ actions, especially when coaches occupy powerful and credible positions. Every time athletes are assessed and coached by a coach or coaching body in a competitive sport setting, ethics cues their behavior and helps them determine if coaches and the organization are behaving fairly. Ethical coaching behaviors are linked to athletes’ commitment, which is why this relationship is positive: athletes who see ethical coaching behaviors are more likely to have a positive internalization of the sport organization.

### 5.2 Trust in coach as the first psychological mechanism

Coach ethical leadership had the strongest direct relationship with trust in coach within the model. The outcome indicates that trust is a key relational consequence of working with a coach from an ethical perspective. This finding is theoretically significant because athletes must be susceptible to the coach’s choices, comments, and commands to trust the coach (Mayer et al., 1995; McAllister, 1995; Rousseau et al., 1998). Coaches are also essential in competitive sport, where athletes frequently rely on them for evaluation, development opportunities, team roles, and emotional support. If coaches act in an ethical manner, then there is even more reason for athletes to feel confident that this dependence will not be abused.

This finding aligns with the results of past studies on leadership and trust, which indicate that fairness, integrity, benevolence, and consistency are all key elements of trust (Mayer et al., 1995; Brown et al., 2005). It also reflects coach–athlete relationship research, which highlights the relational quality, closeness, respect, and commitment that are important to the athlete’s experience (Jowett and Cockerill, 2003; Malloy et al., 2023). The present study builds on this literature by incorporating ethical leadership as a precursor to coach–athlete relationship trust. The study does not only focus on trust as an overall aspect of good relationships, but it also pinpoints one specific area of trust: athletes’ trust in ethical coaching behavior.

The finding also corroborates social learning theory. Athletes build trust not just because coaches are formally appointed or possess technical knowledge. They observe coaches’ decision-making, standard-setting, error correction, opportunity-giving, and handling of pressure. Ethical coaches provide consistent behavioral evidence that they can be trusted. This is especially true for high-performance sport where athletes may feel threatened, compete for resources, feel uncertain about opportunities, or be subject to subjective assessment. Ethical behavior minimizes this uncertainty by demonstrating adherence to rules, rather than acting based on personal favor or interest.

### 5.3 Athlete satisfaction as an evaluative bridge toward commitment

The fact that trust in the coach and athlete satisfaction is positively related suggests that if an athlete trusts their coach, they are more likely to have a positive evaluation of their sport experience. This is in line with the tradition of measuring athlete satisfaction, which has been defined as athletes’ affective and cognitive assessment of coaching, team processes, performance growth, and interpersonal treatment (Riemer and Chelladurai, 1998). The very nature of trust often makes athletes more likely to perceive a coach’s feedback, tough training sessions, and challenging decisions as fair, developmental, and well intentioned.

The positive relationship between athlete satisfaction and organizational commitment also demonstrates the evaluative nature of the link between relational trust and attachment to the organization. This result aligns with organizational commitment theory, more precisely, with the affective commitment perspective which focuses on emotional attachment, identification, and the desire to stay in the organization (Meyer and Allen, 1991; Meyer et al., 1993). Athletes can be non-formal employees of sport organizations but can still create psychological connections with sport teams, clubs, academies, universities, and sport institutes. If they are happy with their experience, they will be more likely to believe that the organization is worthwhile, supportive, and that they want to remain.

These results are in line with the social exchange theory. If athletes feel they are being treated ethically, if they trust the organization, and if they are satisfied with their treatment, they are likely to reciprocate with psychological attachment to the organization (Blau, 2017; Cropanzano and Mitchell, 2005). This exchange process is not a simple transactional exchange but is based on relationship and emotion. When athletes trust their coaches and are happy with their sport experience, they can be induced to feel a sense of obligation, belonging, and loyalty. Therefore, the results indicate that organizational commitment is formed because of a relational exchange process that includes the establishment of trust, satisfaction, and the strengthening of commitment due to ethical leadership.

### 5.4 Serial mediation: linking ethical leadership, trust, satisfaction, and commitment

One of the most salient results of this investigation is the important serial mediation effect. The outcomes revealed that coach ethical leadership is related to organizational commitment in a sequential order: ethical leadership leads to increased trust in the coach, which in turn leads to increased athlete satisfaction, and athlete satisfaction in turn leads to increased organizational commitment. This discovery will contribute a more comprehensive understanding of the importance of ethical leadership in sport organizations.

This is an extension of previous studies, as it combined three previously studied and explored, yet unique, streams of literature: ethical leadership, coach–athlete trust, and athlete satisfaction. Past research has found that ethical leadership could have a positive impact on followers’ attitudes (Brown et al., 2005), that coach–athlete relationships play a key role in athlete outcomes (Jowett and Cockerill, 2003), and that athlete satisfaction is associated with positive sport experiences (Riemer and Chelladurai, 1998). The current study combines these concepts in a process model and demonstrates trust and satisfaction as sequential mechanisms.

This discovery further extends social learning theory and social exchange theory. In the first step, athletes observe ethical coaching behavior and then assess the coach’s credibility based on that behavior (social learning theory). The later stages are explained in terms of social exchange theory: After developing trust with the sport experience, athletes rate their sport experience more positively, and they respond to this in turn by increasing their level of commitment to the organization. The study thus not only presents two theories, but also demonstrates the two theories to be in action at different phases of the same psychological process. Ethical leadership serves a dynamic, moral modeling process and thereafter as a relational exchange process that helps to build commitment.

Not only is it a finding of interest, but it is also practically significant since it indicates that sport organizations cannot rely entirely on ethics codes and values of leadership to engender commitment. Athletes have to experience ethical leadership in a manner that enhances trust and satisfaction. The road to commitment can be compromised when athletes see the coach making ethical judgments but feel that the coach is not fair, caring, and consistent in everyday coaching. Thus, ethics should be a part of daily coaching work rather than confined to formal statements of policy.

### 5.5 Chinese traditionalism as a cultural boundary condition

Chinese traditionalism makes an important contribution with the negative moderation. The findings indicate that coach ethical leadership continues to be positively correlated with trust in the coach at low, mean, and high degrees of traditionalism, but the relationship is weaker with more traditional athletes. This finding indicates that cultural value orientations influence athletes’ perceptions of leadership, thus reinforcing the argument that cultural value orientations do affect athletes’ perceptions of leadership.

Traditionalism is rooted in Chinese culture, which emphasizes respect for authority, acceptance of role obligations in a hierarchical fashion, and deference to senior persons (Farh et al., 1997). In traditional sport organizations, where coaches are already recognized as authority figures, it can be easy for highly traditional athletes to see them as such. Therefore, they may not be as concerned with the coach’s actual ethics, since their cultural values encourage them to obey his or her authority. Conversely, athletes with lower traditionalism may be more influenced by the actual coach behavior when making their judgment about the trustworthiness of the coach. Fairness, transparency, care, and consistency are more concrete behaviors for these athletes that provide evidence of trustworthiness.

This discovery helps to clarify the ethical leadership literature, as it questions the assumption of ethical leadership’s uniformity among followers. Ethical leadership is relevant in all cultural orientations, although its content and depth are related to athletes’ interpretations of authority and moral behavior. This is in line with the literature on cultural leadership research, which reports that individuals’ cultural values can shape their followers’ response to leadership styles (Kirkman et al., 2009). The present study adds to this research by demonstrating that Chinese traditionalism does not change the positive impact of ethical leadership but instead reduces its strength.

It also brings richness to the field of sport leadership studies in China. In the context of Chinese sports, previous research has established that the actions of coaches relate to their athletes’ satisfaction with their team and team-related outcomes (Zhu et al., 2024). The current research builds on these findings by demonstrating the influence of culture on the trust-building effect of ethical coaching. This is relevant because Chinese sports organizations may have a hierarchical structure, and coaches can wield considerable power. The discovery implies that an athlete’s reaction to a coach’s behavior cannot be completely understood without taking into account how they are culturally oriented toward hierarchy and authority.

### 5.6 Moderated serial mediation and conditional process explanation

The indirect effect model with a moderated serial mediation effect indicates that Chinese traditionality moderates the effect of a coach’s ethical leadership on organizational commitment through trust in the coach and athlete satisfaction. The serial indirect effect was largest when the level of Chinese traditionality was low and smallest when the level of Chinese traditionality was high. This finding determines that when coaching values align with cultural values, traditional values weaken the first-stage relationship between ethical leadership and trust in the coach, and this weakening results in a weakening of the second-stage relationship between satisfaction and commitment.

The result builds on the existing literature by demonstrating that cultural values can affect psychological processes as well as actual leadership outcomes. Traditionality does not just alter the link between ethical leadership and trust; it alters the link within the overall process of translating ethical leadership into organizational commitment. The results are in line with conditional process logic, which involves the amount of the indirect effect being moderated by a process at an early stage of an indirect pathway (Hayes, 2018).

The finding is also culturally sensitive in the concept of ethical leadership in sport, as it offers a more nuanced explanation with the mediation of a moderator. Ethical leadership seems more likely to be a behavioral predictor for the lower traditionality group than for athletes who are more traditional, and trust is a stronger mediator between ethical leadership and satisfaction/commitment for the lower traditionality group. For those with greater traditionality, the same route is still important but less influential, suggesting that formal authority and role expectations may also play a partial role in building trust, in the absence of observable ethical conduct. This does not indicate that it is less important for those athletes with a more traditional approach. Instead, it is the psychological mechanism that makes commitment to ethical leadership possible that may vary between cultural value orientations.

### 5.7 Theoretical implications

The present study has a number of theoretical implications. First, it extends the theory of ethical leadership to the competitive sport environment to highlight the relationship between coach ethical leadership and the organizational commitment of athletes. While much research has been conducted on ethical leadership within the workplace, the athletic context presents a unique population, as athletes are located within sport organizations but are not always recognized as employees in the traditional sense. The results indicate that ethical leadership theory can be applied to the athlete population since athletes are equally sensitive to fairness, integrity, moral communication, and principled decision-making.

Second, it enhances research on coach–athlete relationships by providing evidence of trust in the coach as a mechanism between ethical leadership and athlete satisfaction. This builds on the body of literature that has previously been limited to assertions that coaching has a positive impact on athlete outcomes. The findings reveal that ethical behavior is important as it fosters trust, and trust influences athletes’ perceptions of their sport experience.

Third, the study contributes to the conceptualization of organizational commitment research by showing that satisfaction with the sport organization is an important pathway to organizational commitment in sport organizations. The results indicate that affective commitment theory is relevant in the context of competitive sports and demonstrate that affective attachment to organizations is a result of relational and evaluative experiences.

Fourth, the study’s sequential use of social learning theory and social exchange theory is noteworthy. Social learning theory is used to explain how ethical coaching behaviors provide an observable moral cue, which influences trust. Social exchange theory illustrates how trust and satisfaction lead to organizational commitment in terms of positive reciprocal attitudes. This integration offers a more precise theoretical explanation than either theory alone can.

Fifth, the study enriches the study of ethical leadership with a cultural contingency approach by demonstrating that Chinese traditionality affects the ethical leadership process. This contribution is significant because research in sport leadership frequently examines moderators other than culturally based value orientations, such as demographic factors or sport-related moderators. The study reveals the impact of cultural values on athlete perceptions of coaching behavior and the nature of leadership effects.

### 5.8 Practical implications

The results have several practical implications for coaches, sport administrators, and sport organizations. First, ethics education programs need to make ethical leadership a fundamental competency for coaches, not a side issue involving a moral obligation. Coaches need to be trained, educated, and instructed in making fair decisions, communicating in a transparent manner, applying rules consistently, avoiding favoritism, and maintaining the welfare of the athlete. These behaviors are not only good, but also essential to creating trust and commitment.

Second, it should be put in place by sport organizations to facilitate ethical coaching. This may involve clear codes of conduct, open and clear selection processes, grievance procedures, athlete feedback, and regular review of coaching behavior. Restrictions are in place to ensure that the coach’s authority is used responsibly, as athletes rely on coaches for opportunities and evaluation.

Third, coaches should make a point of establishing trust on a daily basis through their behavior. Formal authority will not generate the trust that is needed, particularly among less traditional athletes. Coaches need to be clear about their decisions, give developmental feedback, accept errors, and show concern for the athlete’s health and safety. Such practices can decrease uncertainty and ensure that the athlete feels treated fairly and with support from the coach.

Fourth, athlete satisfaction needs to be tracked as an important measure of organizational wellbeing. The results indicate that satisfaction is strongly associated with commitment. Sport organizations should therefore evaluate the satisfaction of the athletes with the coaching, the training environment, the communication, the clarity of the role, the team climate, and performance development. Prompt identification of dissatisfaction could avert withdrawal, disengagement, or diminished attachment to the organization.

Overall, China’s coach development should be culturally responsive. Athletes vary in their willingness to accept hierarchical authority and the authority of their elders. Assumptions that coaches make regarding how athletes understand authority are not warranted. For sports participants who do not play at the Olympic Games, honesty can be particularly significant in creating trust. The coach must ensure for more traditional athletes that authority is not being misused because athletes may be culturally predisposed to defer to the coach’s authority. Ethical leadership is still required in all areas of traditionality.

## 6 Conclusion

This study investigated the correlations among coach ethical leadership, athletes’ organizational commitment, and the competitive sport context in China. Based on social learning theory and social exchange theory, the study established and verified a moderated serial mediation model, in which trust in coach and athlete satisfaction served as a first-stage mediator and a second-stage mediator, respectively, while Chinese traditionality was a first-stage moderator. All of the proposed hypotheses were supported by the findings. Organizational commitment, trust in coach, and athlete satisfaction were all found to be positively related to coach ethical leadership. The results showed that trust in coach was positively related to satisfaction with coach, and satisfaction with coach was positively related to organizational commitment. The results of the mediation analysis also revealed that the relationship between coach ethical leadership and organizational commitment was significantly explained by trust in coach and satisfaction with coach. Furthermore, the relationship between coach ethical leadership and trust in coach was undermined by the traditionality of coaching, and the serial indirect pathway of trust in coach and athlete satisfaction to organizational commitment was also weakened.

The study has several theoretical contributions. First, it expands the understanding of ethical leadership theory in the competitive sport setting and demonstrates the role of coach ethical leadership in competitive sport organizational commitment. Second, it contributes to the science of coach–athlete relationship research by proving that trust in the coach is an important psychological factor that impacts athlete satisfaction when the coach is ethical. Third, it adds to the research on organizational commitment by suggesting that although athlete satisfaction is an evaluative path, it is also important in the formation of organizational attachment from the perspective of relational trust. Fourth, the integration of social learning theory and social exchange theory indicates that athletes gain their understanding of coaches’ ethical conduct from their learning and interpretation, which provides a foundation for trust, and then experience satisfaction and commitment in response to that trust. Fifth, it adds the traditional culture perspective as the culturally manifested boundary conditions, showing that the traditional culture values of athletes influence the degree of the ethical leadership process.

There are also practical implications of the study. Sport organizations must understand that ethical coaching is not just a moral duty but also an organizational asset. Coaches who behave fairly, consistently, and transparently can enhance the trust and satisfaction of athletes, which can lead to greater commitment to their team, club, academy, and sport organization. Coach development programs should, therefore, contain exercises in ethical decision-making, clear communication, equitable role assignment, athlete wellbeing, and the responsible exercise of authority. Sport organizations should also track the satisfaction of sportspersons and create mechanisms that enable sportspeople to voice concerns without fear of repercussions. Culturally responsive coaching in the Chinese sport environment is particularly significant as athletes may have different levels of deference to authority. While this is true for all athletes, coaches should be more explicit about and demonstrate ethical leadership, as athletes who are less trusting of traditional authority structures may need such reminders.

### 6.1 Limitations

Although this study has contributed in these ways, there are some limitations. First, the cross-sectional design does not allow for making causal statements. The model is theory-based, but future research would benefit from employing time-lagged and/or longitudinal designs to explore change over time in ethical leadership, trust, satisfaction, and commitment. Second, self-reported data was used for the study, which could introduce concerns of common method bias and social desirability. While procedural and statistical remedies were employed, future research could gather data from a variety of sources such as coaches, teammates, and administrators. Third, only competitive athletes from certain cities in mainland China were chosen. Future research should subject the model to testing in other cultural settings, sport systems, and regions to explore the generalizability of the findings. Fourth, Chinese traditionality was selected as the cultural moderator in the study. Other cultural and psychological boundary conditions (e.g., power distance orientation, moral identity, perceived justice, psychological safety, and athlete autonomy) could be explored in future studies. Fifth, the constructs have been considered reflective, and the model has been assessed at the individual level. Multilevel designs may be considered in future research, as coach- and team-level effects are important, particularly given that athletes are frequently nested within teams and training groups.

### 6.2 Future research

This study could be extended in a number of ways. Longitudinally, the investigation could determine if increases in trust, satisfaction, and commitment result from coach ethical leadership over a competitive season. Experimental or intervention-based research studies may evaluate the impact of ethics-based coach training on athlete outcomes. Comparative studies might explore the differences between various types of sport, including individual versus team sports, elite versus non-elite sports, or Chinese versus non-Chinese sport contexts. Future studies could also explore the relationship between athlete satisfaction and organizational commitment with athlete performance-related outcomes such as persistence, training engagement, cooperation, and team cohesion.

Finally, the findings of this study show that coach ethical leadership is a significant predictor of athletes’ organizational commitment, given that it is related to building trust in the coach and to increasing athlete satisfaction. The results further indicate that this process is culture-specific: Chinese traditionality undermines the building process of trust in ethical leadership and the indirect path to commitment. The study establishes a moderated serial mediation model of ethical leadership, trust, satisfaction, commitment, and cultural values to comprehensively explain the relationship between ethical coaching and athlete and organizational outcomes in Chinese competitive sports.

## Data Availability

The original contributions presented in the study are included in the article/supplementary material, further inquiries can be directed to the corresponding author.

## Appendix

**Table A1:** Control variable effects.

Control path	*β*	*t*	*p*	VIF
Gender → TR	0.02	0.51	0.610	1.11
Gender → AS	−0.01	0.33	0.741	1.13
Gender → OC	0.03	0.77	0.441	1.12
Age → TR	0.04	1.08	0.281	1.18
Age → AS	0.03	0.92	0.358	1.17
Age → OC	0.04	1.14	0.254	1.19
City → TR	−0.02	0.54	0.589	1.21
City → AS	0.01	0.29	0.772	1.20
City → OC	−0.02	0.63	0.529	1.22
Sport type → TR	0.05	1.31	0.190	1.09
Sport type → AS	0.04	1.18	0.238	1.10
Sport type → OC	0.05	1.42	0.156	1.11
Organization pathway → TR	0.04	1.18	0.238	1.16
Organization pathway → AS	0.03	0.86	0.390	1.17
Organization pathway → OC	0.04	1.21	0.226	1.18

TR, trust in coach; AS, athlete satisfaction; OC, organizational commitment. All control variables were retained in the robustness model and were nonsignificant.
